# Melatonin’s role in the timing of sleep onset is conserved in nocturnal mice

**DOI:** 10.1038/s44323-024-00013-1

**Published:** 2024-11-01

**Authors:** Pureum Kim, Nicholas Garner, Annaleis Tatkovic, Rex Parsons, Prasad Chunduri, Jana Vukovic, Michael Piper, Martina Pfeffer, Marco Weiergräber, Henrik Oster, Oliver Rawashdeh

**Affiliations:** 1 School of Biomedical Sciences, Faculty of Medicine, University of Queensland, Brisbane, QLD Australia; 2 Queensland Brain Institute, University of Queensland, Brisbane, QLD Australia; 3 Institute of Anatomy 2, Faculty of Medicine, Heinrich Heine University, Düsseldorf, Germany; 4 Experimental Neuropsychopharmacology, Federal Institute for Drugs and Medical Devices, Bonn, Germany; 5 Institute of Neurobiology, Center of Brain, Behavior & Metabolism, University of Lübeck, Lübeck, Germany; 6 Present Address: Australian Centres for Health Services Innovation and Healthcare Transformation, School of Public Health and Social Work, Faculty of Health, Queensland University of Technology, Kelvin Grove, QLD Australia

**Keywords:** Circadian rhythms and sleep, Melatonin

## Abstract

Melatonin supplementation strengthens non‐restorative sleep rhythms and its temporal alignment in both humans and night-active rodents. Of note, although the sleep cycle is reversed in day-active and night-active (nocturnal) mammals, both, produce melatonin at night under the control of the circadian clock. The effects of exogenous melatonin on sleep and sleepiness are relatively clear, but its endogenous role in sleep, particularly, in timing sleep onset (SO), remains poorly understood. We show in nocturnal mice that the increases in mid-nighttime sleep episodes, and the mid-nighttime decline in activity, are coupled to nighttime melatonin signaling. Furthermore, we show that endogenous melatonin modulates SO by reducing the threshold for wake-to-sleep transitioning. Such link between melatonin and SO timing may explain phenomena such as increased sleep propensity in circadian rhythm sleep disorders and chronic insomnia in patients with severely reduced nocturnal melatonin levels. Our findings demonstrate that melatonin’s role in sleep is evolutionarily conserved, effectively challenging the argument that melatonin cannot play a major role in sleep regulation in nocturnal mammals, where the main activity phase coincides with high melatonin levels.

## Introduction

Melatonin secretion follows a robust circadian rhythm with peak levels confined to the nighttime, hence the name “*Hormone of Darkness*”^1^. The circadian master clock in the suprachiasmatic nucleus (SCN) is both the generator of rhythmic pineal melatonin synthesis and release, and a target for melatonin action. The daily nighttime feedback of melatonin on the SCN is part of the resetting mechanism for maintaining intrinsic 24-h physiological rhythms synchronized to geophysical time. The phase resetting properties of melatonin are mediated by melatonin receptor-dependent signaling within the SCN^2–5^, and consequently, although indirectly, influence SCN clock-dependent downstream physiological processes, particularly the sleep/wake cycle^6–8^. In parallel, melatonin also has immediate effects on sleep^9^. The role of melatonin in sleep maintenance and quality has been documented in clinical studies, particularly in elderly and hypertensive patients suffering from low nighttime melatonin levels due to aging or medication^10^. Furthermore, pharmacologically compensating for the loss of endogenous melatonin using melatonin supplements was shown to help increase total sleep duration, improve sleep efficiency, and eliminate sleep rebound following withdrawal from treatment^11,12^. Melatonin’s immediate effects on sleep are accompanied by a dose-dependent lowering of core body temperature, which has been suggested as part of its mechanism of action^13,14^.

The effects of exogenous melatonin on sleep and sleepiness are relatively clear, but its endogenous role in sleep remains poorly understood. It is well-established that the time of sleep onset (SO) is partially dependent on the homeostatic sleep pressure, which progressively accumulates during wakefulness^15^, and that the melatonin rhythm is closely associated with the timing of sleep and sleep propensity in humans^16–19^. This may also be the case in nocturnal rodents according to a comprehensive literature analysis integrating data from rodent and human electroencephalography-confirmed sleep studies^20^. Particularly, it was noted that the sleep-promoting effect of melatonin was most evident when melatonin treatment temporally coincided with high sleep pressure. This raises the question if the rise of nighttime endogenous melatonin levels, which aligns in phase with the time of SO, is causative or correlative. We hypothesize that endogenous melatonin is instrumental in modulating the timing of SO, which we here investigate in nocturnal mice.

Hindering basic research efforts into understanding the neurobiological mechanism underlying the soporific effects of melatonin is the misconception that sleep in nocturnal melatonin-proficient mice is limited to the light phase. Our study shows that, also in mice, SO and a significant portion of the daily sleep pensum are in fact confined to the dark phase. Furthermore, the timing of SO aligns with the previously reported rise in nocturnal pineal melatonin levels in mice^21^ and is shifted in melatonin receptor deficient mice. We also establish that the sleep-promoting effect of melatonin is melatonin receptor dependent. The sleep-promoting effects of melatonin can be parsimoniously explained by its actions on the SCN, since melatonin inhibits SCN neuronal activity, or downstream of SCN-modulated cortical and behavioral activity.

## Results

### Running-wheel-based immobility-defined sleep onset latency is advanced in MT_1/2_^+/+^ mice

**Fig. 1:**
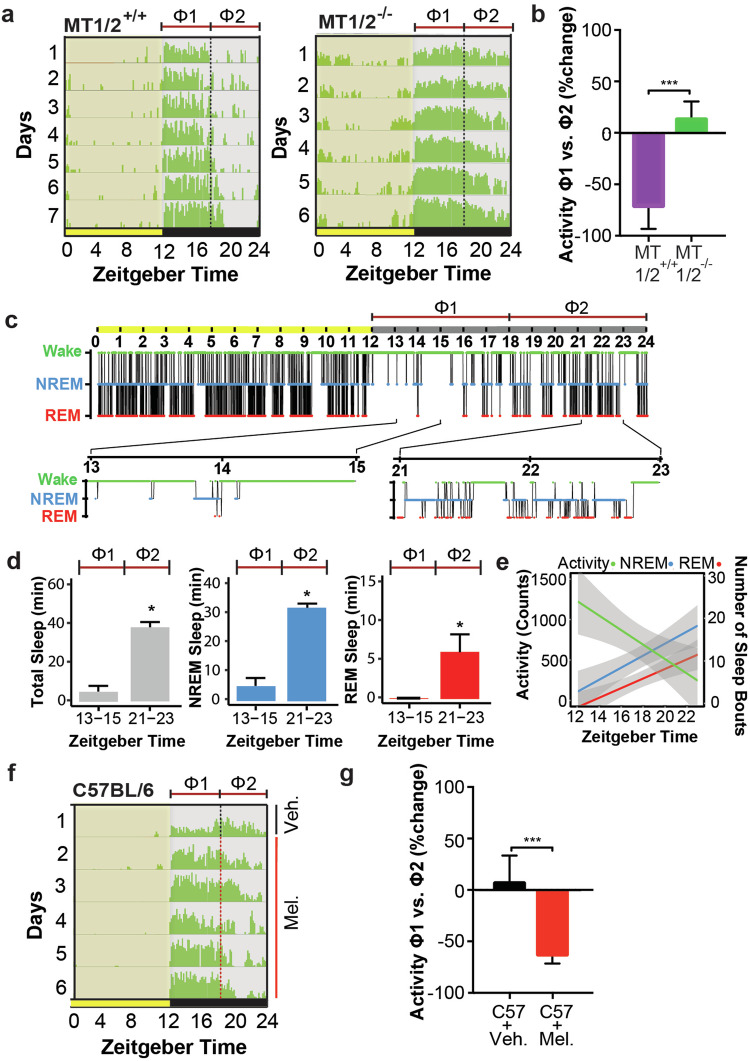
Endogenous melatonin modulates nighttime activity and the timing of nocturnal sleep-onset. **a** Average running wheel activity profiles for MT_1/2_^+/+^ (*n* = 5) and MT_1/2_^−/−^ mice (*n* = 7). The vertical line divides the nighttime into 6-h bins, and Φ1 and Φ2 indicate the 1st and the 2nd half of the nighttime, respectively. **b** Comparison of the mean difference in activity between Φ1 and Φ2 across both genotypes (MT_1/2_^+/+^; *p* < 0.0001, and MT_1/2_^−/−^ *p* > 0.05, Student’s *t*-Test). **c** Representative hypnogram showing NREM and REM sleep stages for MT_1/2_^+/+^ mice. Below are high-resolution hypnograms corresponding to the early dark phase (ZT13-ZT15 of Φ1) and the late dark phase (ZT21-ZT23 of Φ2). **d** Quantification of total sleep (), NREM sleep () and REM sleep () during the early and late night (ZT13-ZT15 and ZT21-ZT23, respectively) in MT_1/2_^+/+^ mice (*n* = 6). **e** Distribution of activity (), NREM (), and REM sleep () during the dark phase in MT_1/2_^+/+^ mice (*n* = 6). **f** Average running wheel activity profile for C57BL/6 mice (*n* = 6), treated with vehicle (veh.) on day 1 and melatonin (mel.) on days 2–6. Yellow and gray sections show light and dark phases, respectively. The actograms show wheel revolutions/10 min bins. **g** Comparison of the mean percent change in nocturnal activity between Φ1 and Φ2 between veh. and mel. treated C57BL/6 mice (C57BL/6 mice during Φ2 veh. (day 1) and mel. (day 6); *p* < 0.005, Student’s *t*-Test, *n* = 5). The data are presented as means ± S.E.M. * and *** indicate *p* < 0.05 and *p* < 0.0001, respectively. ns stands for *p* > 0.05.

The profile of nocturnal running-wheel (RW) activity in mice is shaped by both sleep homeostasis (process S) and the circadian clock (process C). For example, sleep-onset is determined by sleep history (accumulated sleep pressure) and biological time (circadian pressure). Since melatonin is an output of the biological clock and has sleep-inducing (hypnotic) properties, we hypothesized that melatonin can influence the nocturnal activity profile by timing SO. The data show that nocturnal RW activity of MT_1/2_^+/+^ mice declined abruptly after ~6-h (ZT18) into the dark phase (Fig. 1a, b). Overall, RW activity during the 2nd half of the night (ZT18-24; Φ2) decreased by 82.0 ± 12.6% (distance traveled) compared to the average activity in the 1st half of the night (ZT12-18; Φ1) when endogenous melatonin is at its nadir^21–23^. Conversely, wheel-running activity in melatonin receptor deficient (MT_1/2_^−/−^) mice extended across most of the night (Fig. 1a, b).

To confirm that the absence of RW activity during the 2nd half of the night in MT_1/2_^+/+^ mice indeed represents sleep, we assessed electroencephalography (EEG) defined sleep. The data show a clear difference in the distribution of wakefulness between Φ1 and Φ2, with most of the nocturnal sleep confined to Φ2 (Fig. 1c–e).

Like MT_1/2_^−/−^ C3H/HeN mice, C57BL/6 mice showed extended nocturnal RW activity. However, unlike MT_1/2_^−/−^ mice, C57BL/6 mice are pineal melatonin deficient but express melatonin receptors and thus can respond to exogenous melatonin. To test whether immobility-defined SO (Fig. 1f, g) can be advanced by melatonin, we treated C57BL/6 mice for 6 days with melatonin in the drinking water provided at mid nighttime. Indeed, timed melatonin intake significantly reduced nocturnal wheel-running activity during the 2nd half of the night (Φ2) by 62.7 ± 8.8% compared to the 1st 6-h of darkness (Φ1) (Fig. 1f, g).

### Home cage immobility-defined sleep timing is advanced in MT_1/2_^+/+^ mice

**Fig. 2:**
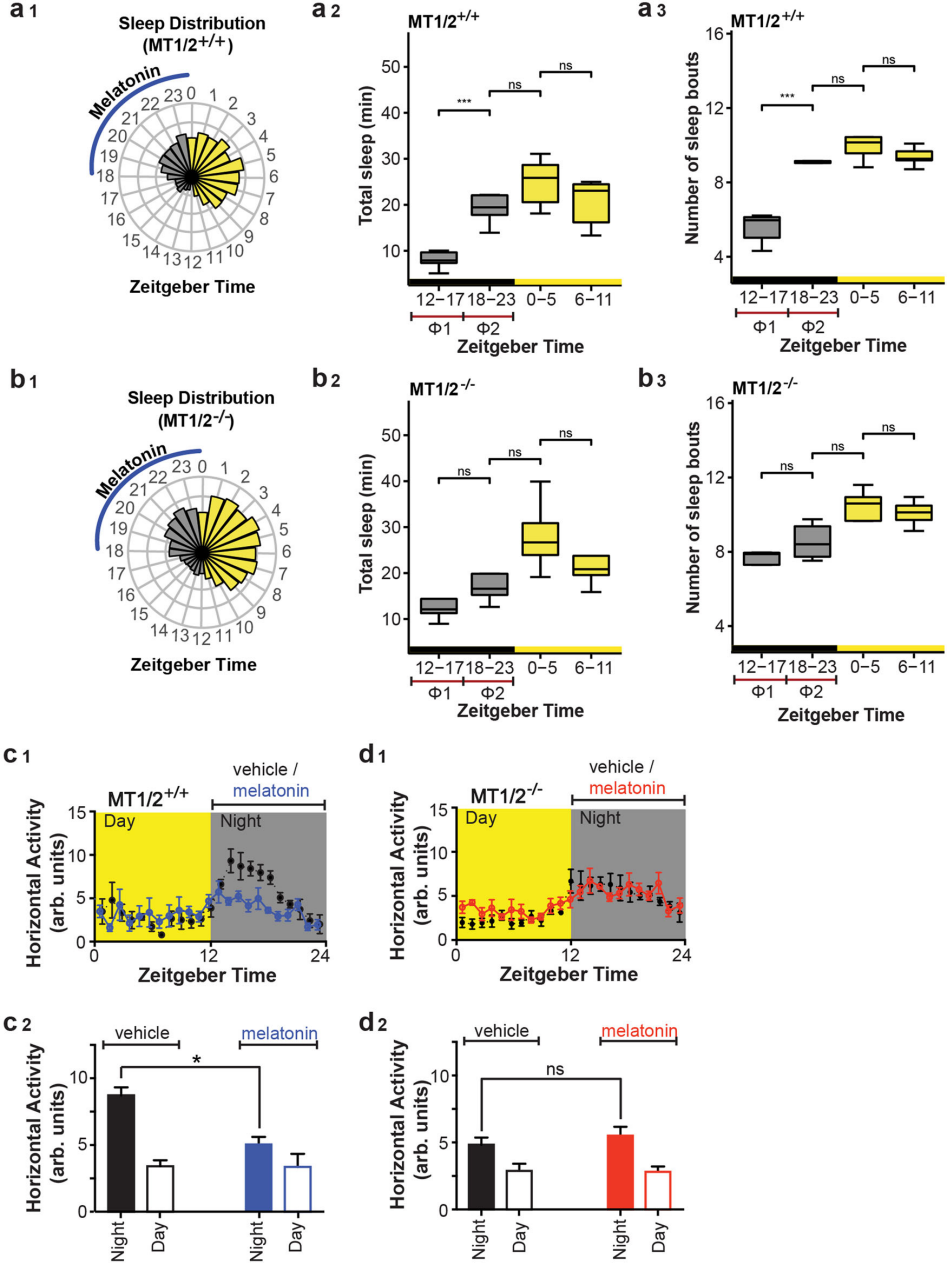
Comparison of immobility-defined sleep parameters between melatonin receptor proficient and deficient mice. **a**_**1**_ Circular bar plot showing the 1-h averages of the amount of infrared-based immobility-defined sleep distributed across a 12:12 h LD cycle in MT_1/2_^+/+^ mice (*n* = 7). ZT stands for Zeitgeber Time, and the blue line indicates the phase of peak endogenous melatonin levels. **a**_**2**_ A box-and-whisker blot for the total amount of sleep for MT_1/2_^+/+^ mice (one-way ANOVA followed by pairwise Student’s *t*-Test, *p* < 0.005). **a**_**3**_ A box-**a**nd-whisker blot for the number of sleep bouts (one-way ANOVA followed by pairwise Student’s *t*-Test, *p* < 0.05) for MT_1/2_^+/+^ mice. **b**_**1**_ Circular bar plot showing 1-h averages of the amount of sleep distributed across a 12:12 h LD cycle in melatonin receptors deficient mice (MT_1/2_^−/−^, *n* = 8). **b**_**2**_ A box-and-whisker blot for the total amount of sleep for MT_1/2_^−/−^ mice (one-way ANOVA followed by pairwise Student’s *t*-Test, *p* > 0.05). The total amount of sleep during the 2nd half of night (Φ2) is comparable with the amount of sleep during the light phase. **b**_**3**_ A box-and-whisker blot for the number of sleep bouts for MT_1/2_^−/−^ mice (one-way ANOVA followed by pairwise Student’s *t*-Test, *p* < 0.05). **c**_**1**_, **d**_**1**_ Horizontal (home-cage) locomotor activity profiles for mice (MT_1/2_^+/+^ and MT_1/2_^−/−^) treated with vehicle or melatonin in drinking water. Comparison of day and night activity for MT_1/2_^+/+^ (**c**_**2**_, *n* = 7) and MT_1/2_^−/−^ (**d**_**2**_, *n* = 8) mice between treatment groups (Student’s *t*-Test, *p* < 0.05). The data are presented as means ± S.E.M. * and *** indicate *p* < 0.05 and *p* < 0.0001, respectively. ns stands for *p* > 0.05.

Wheel-running activity is considered a complex behavior, reflecting several underlying processes in addition to spontaneous home cage activity^24^. To validate and extend the RW data, we assessed high-throughput infrared-based horizontal activity data from MT_1/2_^+/+^ and MT_1/2_^−/−^ mice to determine whether immobility-defined sleep in the absence of a stimulant, here a RW, is modulated by endogenous melatonin (Fig. 2). MT_1/2_^+/+^ mice show significantly more inactivity-based total sleep and number of sleep bouts during the 2nd half of the night compared to the first 6-h of darkness (Fig. 2a_1_–a_3_). Although MT_1/2_^−/−^ mice experience more inactivity-based sleep during the 2nd half of the night compared to the first 6-h of darkness (Fig. 2b_1_–b_3_), this was not significant. The earlier decline in energy expenditure in MT_1/2_^+/+^ mice compared to MT_1/2_^−/−^ mice (Fig. S1) further corroborates these findings. A comparison across genotypes revealed a significant difference in the amount of inactivity-based total sleep between Φ1 (ZT12-17) and Φ2 (ZT18-23) (Φ1 vs Φ2: +1.53-fold in MT_1/2_^+/+^ vs. +0.416-fold in MT_1/2_^−/−^ mice, *t*-test, *p* < 0.001), which is also visible in the 24-h activity profiles for both genotypes (Fig. 2c_1_, d_1_). Similarly, the number of inactivity-based sleep bouts in the first 6 h of the dark phase (Φ1) versus the second half of the nighttime (Φ2) between genotypes was significantly different (ZT12-17 vs ZT18-23: +0.8-fold in MT_1/2_^+/+^ vs. +0.0625-fold in MT_1/2_^−/−^ mice, *t*-test, *p* < 0.001).

### Suppression of nocturnal activity by melatonin is receptor dependent

To determine whether melatonin per se is a potent modulator of nighttime activity, we assessed the effect of exogenous melatonin on the amplitude of the sleep/wake rhythm. Exogenous melatonin supplementation in drinking water presented at the beginning of the night, when endogenous melatonin levels are low in MT_1/2_^+/+^ mice, significantly reduced nocturnal activity, resulting in a dampened sleep-wake rhythm (amplitude of the sleep-activity rhythm: vehicle vs. melatonin treatments; 2860.4 ± 535.4 vs. 1231.8 ± 747.5, *p* < 0.05, Fig. 2c, d and Fig. S2). The absence of a response to exogenous melatonin in MT_1/2_^−/−^ (Fig. 2c, d and Fig. S2) suggests that the nighttime suppression of nocturnal activity by melatonin is melatonin receptor-dependent. Importantly, the lack of response of MT_1/2_^−/−^ mice to melatonin was not due to genotypic differences in the amount of melatonin intake per body weight (Fig. S3), which further supports a melatonin-receptor dependent effect.

### Melatonin administration enhances light-induced behavioral sleep via melatonin receptor-dependent signaling

**Fig. 3:**
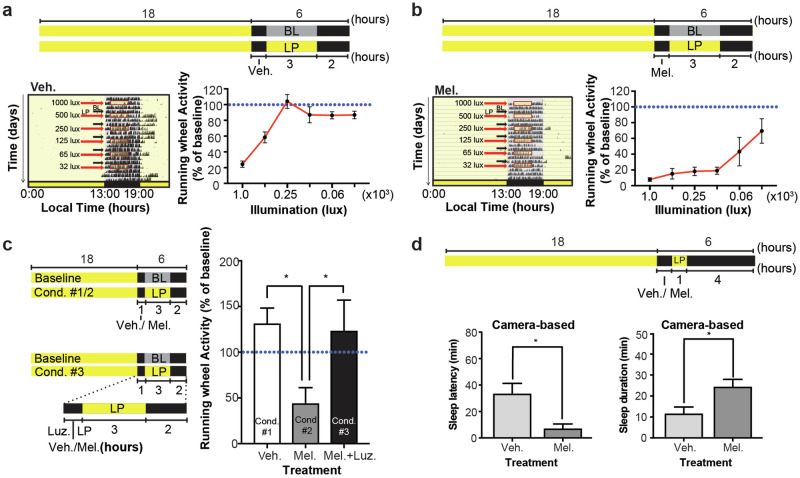
Melatonin potentiates light-induced suppression of locomotor activity and sleep induction. **a**, **b** (Top) MT_1/2_^+/+^ mice (*n* = 8) pre-treated (i.p. injections) with vehicle (veh.) or melatonin (mel.) were exposed to a 3-h light pulse (LP) of different illuminance during the night. (Left) Representative actograms for vehicle and melatonin injected mice exposed to a LP every 3rd day. (Right) Quantification of the activity data. One-way ANOVA for illuminance of the vehicle treated mice, *F*_(5, 30)_ = 16.85, *p* < 0.0001. One-way ANOVA for illuminance of the melatonin treated mice, *F*_(5, 18)_ = 4.843, *p* _< _0.005. Two-way ANOVA for interaction (treatment vs. illuminance), *F*_(5, 48)_ = 5.184 *p* < 0.00005, illuminance, *F*_(5, 48)_ = 13.48 *p* < 0.0001, and for treatment, *F*_(1, 48)_ = 86.09, *p* < 0.0001. The blue horizontal line corresponds to the baseline activity (BL). **c** (Left) Experimental protocol for three conditions (cond.); mice injected with veh. (*n* = 6), mel. (*n* = 6), or luzindole (luz) and mel. followed by a 3-h LP (65 lux). (Right) Quantification of photosomnolence in MT_1/2_^+/+^ mice. Melatonin enhanced light-induced immobility-defined sleep, and which was blocked when mice were pre-treated with luz. (Student’s *t*-Test, *p* < 0.05). **d** Quantification of video-based SO (left) and sleep duration (right) in MT_1/2_^+/+^ mice entrained to an 18:6 h LD cycle for mice injected (i.p.) with either veh. (*n* = 9) or mel. (*n* = 9) followed by a 1-h LP of 65 lux (Student’s *t*-Test, *p* < 0.05). * Indicates *p* < 0.05. The data are presented as means ± S.E.M.

Considering that in MT_1/2_^+/+^ mice (i) the profile of the sleep-wake rhythm is melatonin-receptor dependent, (ii) that the time when nocturnal activity starts to decline and sleep episodes increase is melatonin receptor-dependent, and (iii) that nighttime melatonin treatment suppresses nocturnal activity, we hypothesized that the circadian signal melatonin suppresses wakefulness. A model by which the circadian process C could set the phase of the SO time. To test this hypothesis, we investigated the sleep-inducing (hypnotic) effects of exogenous melatonin at different levels of sleep propensities “sleepiness”, which we modulated using light i.e., does melatonin increase the probability for the mouse to fall asleep? Utilizing the hypnotic properties of light (photosomnolence), we measured the efficiency of melatonin to induce RW-based immobility-defined sleep at different illuminance. We found that light of 65 lux or less, which is ineffective in inducing immobility-defined sleep in MT_1/2_^+/+^ mice (Fig. 3a), became hypnotic when combined with low-dose intraperitoneal melatonin injections (Fig. 3b). To confirm that the effect of melatonin was mediated via melatonin receptor-dependent signaling, we repeated the experiment after pre-treating mice with the MT_1/2_ receptor antagonist, luzindole. Inhibiting melatonin receptor-dependent signaling blocked the additive effect of melatonin on photosomnolance (Fig. 3c). To visually confirm that the mice were asleep when off the RW, we simultaneously video tracked mouse behavior. The data were also used to score SO latency and sleep duration. Mice injected with melatonin 30 min prior to the light pulse (LP) (65 lux) fell asleep significantly earlier and slept for longer (Student’s *t*-test, *p* < 0.05) (Fig. 3d), confirming the sleep-wake modulating effect of melatonin.

### Endogenous melatonin receptor-dependent signaling enhances the hypnotic effect of light

**Fig. 4:**
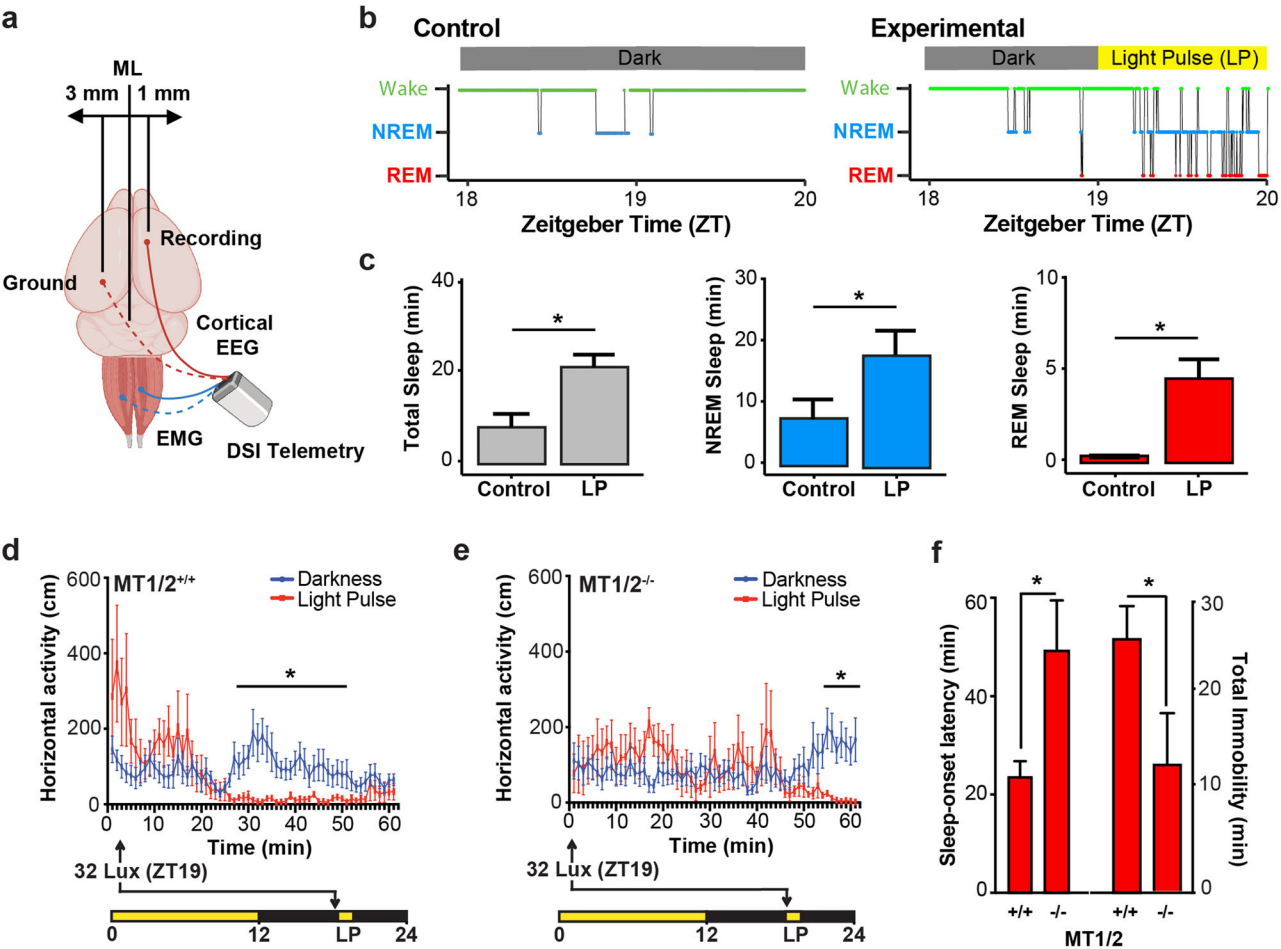
The effect of melatonin on photosomnolence is dependent on melatonin receptor signaling. **a** Illustration showing the placement of EEG and EMG recording electrodes. The image was created with BioRender.com. **b** Representative hypnogram showing darkness control (left) and light-induced (right) NREM and REM sleep during the 1-h light pulse (LP; 65 lux) for a MT_1/2_^+/+^ mouse maintained under a 12:12 h LD cycle. **c** Quantification of total sleep (gray), NREM sleep () and REM sleep () under darkness and LP conditions. **d** MT_1/2_^+/+^ mice (*n* = 6) exposed to a 1-h LP at ZT19 () versus control mice exposed to darkness only (). Light-pulsed mice showed a significant effect of light on infrared-based locomotor activity (two-way ANOVA, *p* < 0.05). **e** MT_1/2_^−/−^ mice (*n* = 6) exposed to a 1-h LP at ZT19 () versus control mice exposed to darkness only (). Light-pulsed mice showed no significant effect of light on infrared-based locomotor activity (two-way ANOVA, *p* > 0.05). **f** Comparison (Student’s *t*-Test, *p* < 0.05) of sleep onset latency and total immobility following a LP between MT_1/2_^+/+^ and MT_1/2_^−/−^. MT_1/2_^+/+^ mice exposed to darkness were active for the duration of the recording (ZT19-ZT20). MT_1/2_^+/+^ mice exposed to a LP fell asleep within 22 ± 5 min compared to 48 ± 6 min in MT_1/2_^−/−^ mice (Student’s *t*-Test, *p* < 0.05). Sleep onset latency was identified as the time from ZT19 until the first sleep episode (60 s or longer of immobility). MT_1/2_^+/+^ mice exposed to a LP accumulated 25 ± 8 min of sleep compared to 8 ± 4 min of sleep in MT_1/2_^−/−^ mice (Student’s *t*-Test, *p* < 0.05). The data are presented as means ± S.E.M. and * indicates *p* < 0.05.

Using a natural photoperiod (12:12 h LD cycles) under which the endogenous melatonin rhythm for MT_1/2_^+/+^ mice has been well characterized^22,25^, we investigated the timing of SO (sleep onset latency) in relation to the rise of nocturnal melatonin levels (ZT17-18)^21^. As a proof of concept, we first established that a LP of 65 lux, which has no sleep-inducing effects at the beginning of the dark phase, can induce NREM and REM sleep episodes in MT_1/2_^+/+^ mice when applied in the late dark phase (Fig. 4a–c). Using a high-throughput infrared-based home cage activity monitoring system we subjected MT_1/2_^+/+^ mice to a 65 lux LP at ZT19, when endogenous melatonin levels peak^21–23^. The 1-h LP induced sleep, as previously defined^26,27^, within 22 ± 5 min (Fig. 4d, f). MT_1/2_^−/−^ mice also showed a significant response to the 65-lux LP (Fig. 4e, f), but compared to MT_1/2_^+/+^ mice, light-induced behavioral SO latency was significantly delayed and thus, induced less immobility-defined sleep (Fig. 4f; Student’s *t*-Test, *p* < 0.05). This result implies that the effect of light on behavioral sleep induction is phase-dependent and modulated by endogenous melatonin in a melatonin receptor-dependent manner.

### Melatonin modulates substrates of the circadian-sleep circuitry

**Fig. 5:**
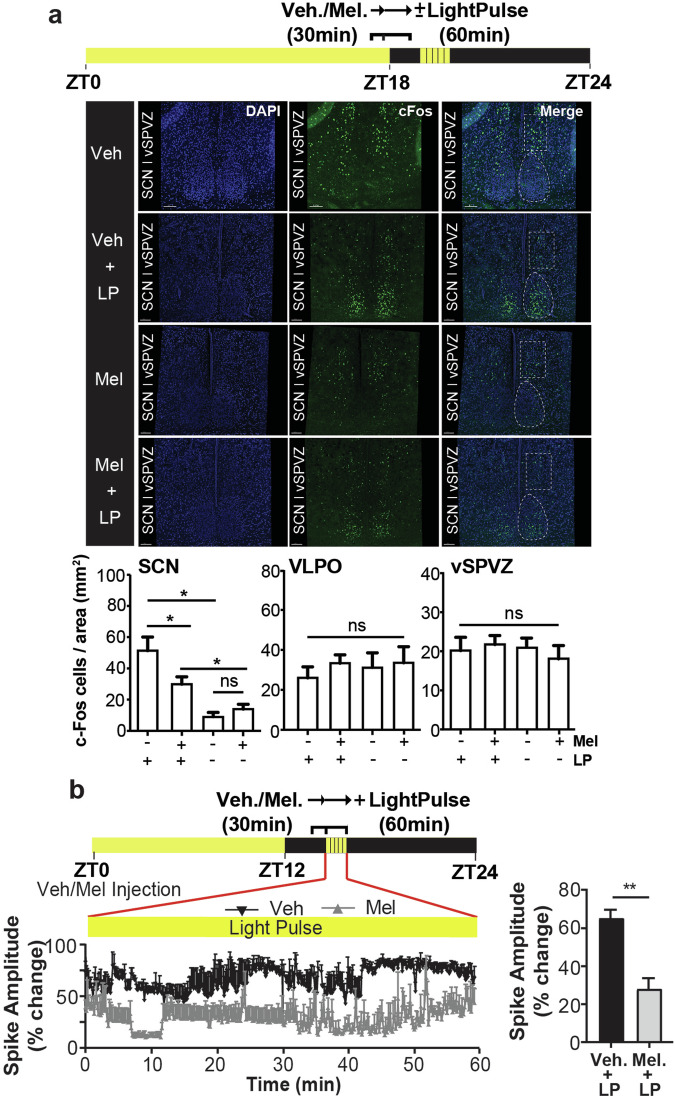
Melatonin differentially inhibits light induced SCN activation. **a** Representative confocal images of cFos-protein expression in the SCN, and vSPVZ of MT_1/2_^+/+^ mice injected with vehicle (veh.; *n* = 4) or melatonin (mel.; *n* = 4), 30 min before a 1-h light pulse (LP; 65 lux). (Bottom) quantification of the cFos signal in the SCN, vSPVZ, and VLPO. Melatonin treatment inhibits light-induced cFos protein expression in the SCN (one-way ANOVA for the effect of melatonin on light-induced cFos expression in the SCN, *F*_(3, 18)_ = 12.45, *p* < 0.0001) with no significant change in cFos protein expression in the vSPVZ (one-way ANOVA, *F*_(3, 20)_ = 0.91, *p*_>_0.8) and VLPO (one-way ANOVA, *F*_(3, 20)_ = 0.166, *p* > 0.7). **b** (Left) Representative SCN neuronal firing during a 60 min. LP for veh. or mel. injected mouse. (Right) Comparison of the 60 min. average percent change in SCN neuronal spike amplitude between veh. and mel. treated MT_1/2_^+/+^ mice (*n* = 3). The data are presented as means ± S.E.M. * and ** indicate *p* < 0.05 and *p* < 0.05, respectively. ns stands for *p* > 0.05.

Next, we investigated the response of neuronal substrates of behavioral sleep induction and sleep regulation to melatonin, specifically, the SCN and the ventral subparaventricular zone of the hypothalamus (vSPVZ)^28,29^. For this purpose, we used the proto-oncogene product (cFos) as a tool to map neuronal activity^28,30,31^. The vSPVZ is known to integrate retinal and SCN input^32–34^, and when activated, induces sleep^28,34^. It is well documented that melatonin inhibits SCN neuronal activity which, considering the inhibitory SCN-vSPVZ communication, suggests that melatonin could modify sleep timing. Indeed, we found that in the SCN, melatonin inhibited light induced cFos-protein expression (Fig. 5a) and reduced light-evoked changes in the amplitude of SCN neuronal spikes (Student’s *t*-test, *p* < 0.005) (Fig. 5b). Exogenous melatonin alone did not alter cFos protein expression in the vSPVZ nor in the ventrolateral preoptic nucleus (VLPO) of the anterior hypothalamus (Fig. 5a).

### Hippocampal rhythms in signaling follows the sleep-wake profile

**Fig. 6:**
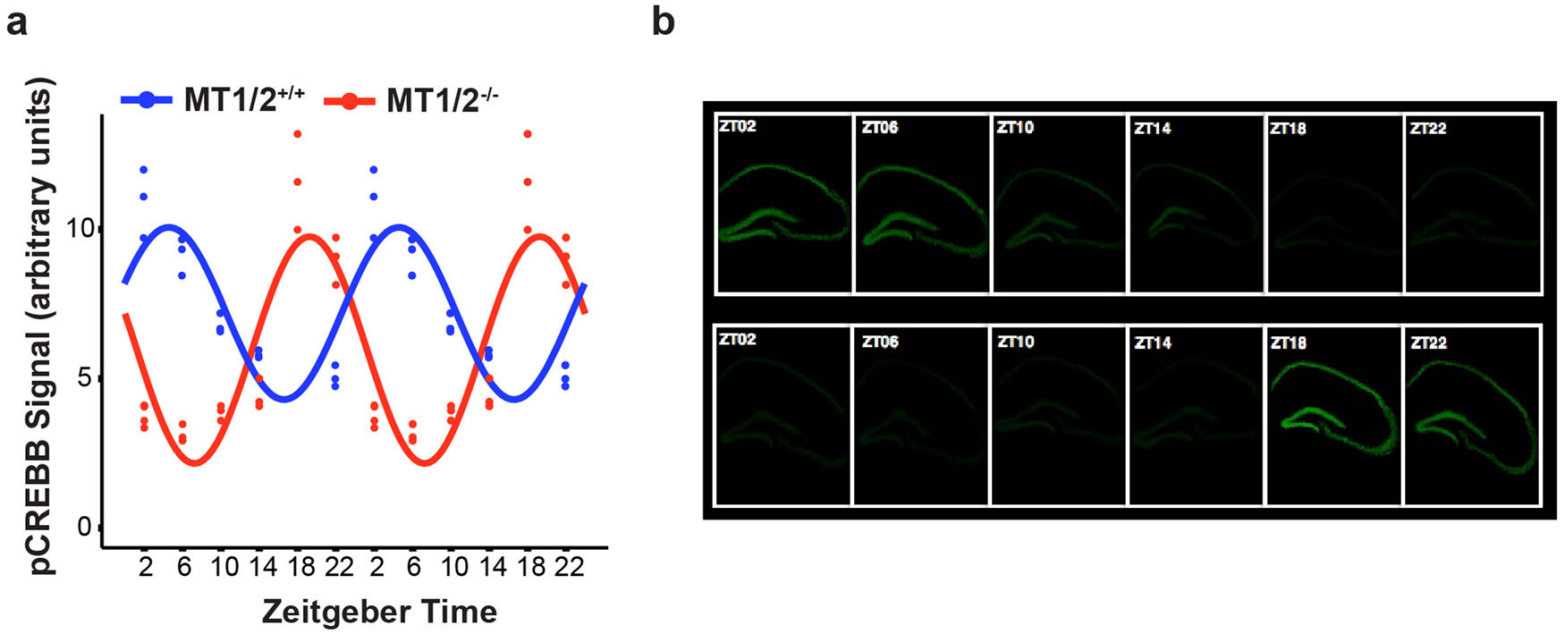
Melatonin receptor signaling sets the phase of the hippocampal pCREB “rhythm”. **a** The diurnal pCREB rhythm is phase advanced in MT_1/2_^−/−^ compared to MT_1/2_^+/+^ mice. For better visualization of the rhythms and their phase relationship, the 24-h data points are double plotted. **b** representative time series of pCREB signal (green) in the dorsal hippocampus of MT_1/2_^−/−^ and MT_1/2_^+/+^ mice. Cosinor analysis was applied to the 24-h dataset.

Considering the differences in the distribution and shape of consolidated wakefulness and sleep across the 24-h day/night cycle and the earlier SO in MT_1/2_^+/+^ mice compared to MT_1/2_^−/−^ mice suggests that rhythms in physiology and behavior (e.g., the time-of-day for optimal learning and memory formation) must also be shifted in both genotypes. Here we tested whether melatonin receptor-dependent differences in the phase of the SO are associated with a shift in the rhythmic profile of hippocampal signaling. We found that the earlier SO in MT_1/2_^+/+^ mice was also reflected in the rhythmic hippocampal phosphorylation of the memory-relevant transcription factor CREB (pCREB). In MT_1/2_^+/+^ mice, the peak of the pCREB rhythm occured at ZT4.65 (cosinor analysis), which was significantly advanced compared to MT_1/2_^−/−^ mice (peak: ZT20.24, cosinor analysis) (Fig. 6a, b).

## Discussion

Melatonin is often referred to as a sleep hormone. Unquestionably, melatonin supplementation does have sleep-promoting effects^20,35–38^, and sleep quality improves when the circadian system is aligned with rhythmic circulating melatonin^39,40^. The latter suggests an association between endogenous melatonin and the timing of sleep^19,40,41^. In our mouse strain, nocturnal melatonin-receptor-dependent signaling occurs during the second half of the night^22,23,25^, when sleep pressure has significantly built up and the circadian drive for wakefulness (process C; circadian alerting signal) is expected to have dropped compared to the beginning of the night. Consequently, we provide evidence that this signaling effectively contributes to regulating SO and shaping the daily sleep-wake or rest-activity profile^42–44^. This is clearly reflected in the nocturnal activity pattern of MT_1/2_^+/+^ when compared to MT_1/2_^−/−^ mice in our study, and the actograms of published work using the same mouse strain^45–48^, where nighttime activity is predominantly restricted to the 1st half of the night, when melatonin levels in these mice are naturally low^22,25,49^. The extended nighttime activity in MT_1/2_^−/−^ is corroborated by EEG/EMG-based vigilance state analysis^50^, showing MT_1/2_^−/−^ spent more time awake compared to MT_1/2_^+/+^ mice. The decline in activity around midnight in MT_1/2_^+/+^ coincides with the published nighttime rise of endogenous melatonin^22,23,25^—which we show marks the onset of increased REM and NREM sleep episodes.

Interestingly, in MT1 receptor knockout mice, an increase in nighttime EEG/EMG-based NREM sleep also starts in the middle of the dark phase, when melatonin levels rise, which supports a role for melatonin receptor-dependent signaling, specifically, MT2 receptor dependent signaling, in timing sleep^50^. However, this study did not report on the characteristic differences in EEG/EMG-based sleep-wake profiles of MT_1/2_^+/+^ mice observed in our study. One possible explanation might be the reduced post-surgical recovery time of only 6 days^50^ as opposed to 14 days in our study. Furthermore, our EEG/EMG-based recordings started during the active phase (nighttime) as opposed to the beginning of the light phase^50^ to avoid interfering with sleep during the resting period.

**Fig. 7:**
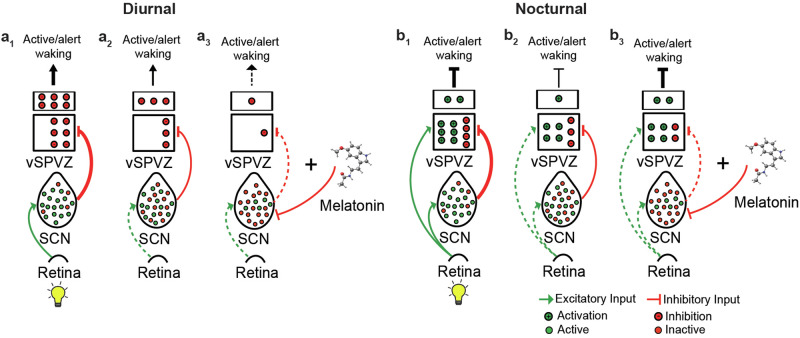
Modeling the evolutionarily conserved role of melatonin on sleep in diurnal and nocturnal species. **a**_**1**_ In diurnal species, the activity of the vSPVZ is predominantly regulated by the SCN. During the day, when the SCN is active (prevalent [green] neurons), the vSPVZ (predominantly inhibitory) is strongly inhibited, decreasing the inhibitory tone of the vSPVZ, resulting in increased alertness. **a**_**2**_ During the night, when the SCN output is relatively neutral (equal number of silent [red] and active [green] neurons), the vSPVZ is less inhibited, increasing the inhibitory tone of the vSPVZ, resulting in reduced alertness. **a**_**3**_ melatonin inhibits the SCN further (predominantly silent [red] neurons), further reducing the inhibitory tone of the vSPVZ, resulting in additionally reduced alertness (facilitating sleep). **b**_**1**_ In nocturnal species, the activity of the vSPVZ is regulated by the SCN and the retina. During the day, light activates the SCN (prevalent [green] neurons) and the vSPVZ (predominantly excitatory). As a result, the excitation of the vSPVZ overcomes the inhibition by the SCN, resulting in a strong positive excitatory tone and inhibiting alertness. **b**_**2**_ During the nighttime, the excitatory tone of the vSPVZ is regulated by the SCN only. A reduced nighttime SCN inhibitory tone and the absence of light-induced vSPVZ activation results in a net decrease in vSPVZ excitatory tone, promoting wakefulness. **b**_**3**_ Similar to diurnal mammals at night, melatonin further inhibits the SCN’s inhibitory tone, resulting in the vSPVZ’s excitatory to inhibitory tone ratio increasing and enhancing the inhibition of alertness. The bold lines indicate strong signal strength, and hashed lines refer to a weak or absent signal. Modified from ref. ^28^.

Because melatonin-deficient C57BL/6 mice differ from melatonin-proficient C3H/HeN (MT1/2) mice on various genetic, anatomical, and behavioral levels^51,52^, we included melatonin-proficient C3H/HeN mice that are deficient for melatonin receptors 1 and 2 (MT_1/2_^−/−^)^53^. Like the C57BL/6 strain, MT_1/2_^−/−^ mice are active across much of the night. The earlier onset of sleep in MT_1/2_^+/+^ mice could be explained by the wake-inhibiting (sleep-facilitating) properties of melatonin^28^ (Fig.7), likely further potentiated by the increased sleep pressure that builds up with time awake (Fig. S4).

Studies in humans and rats indicate that melatonin may be involved in the induction or regulation of sleep. Putative endogenous hypnotic substances can affect sleep similarly to “physiological sleep pressure”^54^. Here, we show that by supplementing pineal melatonin-deficient C57BL/6 mice with exogenous melatonin, nocturnal activity consolidates within the first half of the night, which is similar to melatonin-proficient MT_1/2_^+/+^ mice and reflects a change in SO timing. To test whether melatonin can regulate the SO time by modulating its sleep-inhibiting effects via the SCN, we used light in a dose-dependent manner to gradually suppress the circadian alerting signal, which phase-dependently can counterbalance sleep pressure^55–57^.

The efficiency of light to induce sleep in mice decreases with illuminance^58–61^ and is photoperiod dependent^62^. Thus, light can modulate vigilance states by modulating the circadian drive for wakefulness. To test whether the effects of light on sleep can be potentiated by melatonin, we combined exogenous melatonin treatment with different levels of illuminance and measured sleep induction per established criteria^26,27^. In our study, exogenous melatonin enhanced the sleep-inducing effects of light in a dose-dependent manner. By blocking melatonin receptor signaling, we confirmed that this effect is receptor dependent. To assess whether the same applies to endogenous melatonin, we compared the efficiency of light-induced sleep between MT_1/2_^+/+^ and MT_1/2_^−/−^ mice during the second half of the night when melatonin levels peak in both genotypes. Our results closely resemble the acute effect of exogenous melatonin on EEG/EMG-based sleep when applied at the beginning of the night^20^ when endogenous melatonin is low. We conclude that endogenous melatonin is a significant player in structuring nighttime activity profiles in MT_1/2_^+/+^, which parallel the genotypic differences in immobility-defined SO times. Variations in the efficiency of light-induced immobility-defined sleep induction and circadian differences in light sensitivity^63,64^ between genotypes are additional factors that could have influenced the results and thus warrant further investigation.

Research on mice aimed at understanding how melatonin might be used as a drug in humans has often utilized high doses, 100 mg/kg^65,66^ – 200 mg/kg^67,68^, as reviewed by David Kennaway^69^. In contrast, our study uses a significantly lower but still pharmacological dose of melatonin (3 mg/kg), which is consistent with the concentrations and administration routes used in studies assessing sleep and circadian rhythms. In studies examining the acute EEG-based sleep-inducing effect of melatonin in rats^20^, a dose of 3 mg/kg was found to have a hypnotic effect lasting ~60 min, which aligns with our findings using non-EEG-based methods in mice. Interestingly, at this dose, melatonin does not exhibit a sleep inducing effect when administered during the light phase^70^. Indeed, the hypnotic effect of melatonin is dependent on the environmental lighting conditions^20^.

The cFos expression analysis within the brain’s sleep regulatory centers proposes that melatonin’s function on sleep facilitation is communicated via its inhibitory action on the SCN and subsequently on the vSPVZ^28^. Alternatively, the active inhibition of SCN activity and the simultaneous activation of the brain’s sleep centers could collectively contribute to sleep initiation and maintenance. According to the current knowledge on the neural circuitry of sleep regulation in nocturnal mice, activation of the SCN by light indirectly inhibits downstream SCN targets, particularly the vSPVZ^28^. However, light also activates vSPVZ neurons via direct input from the retinohypothalamic tract. Collectively, these suggest that the inhibitory tone from the SCN onto the vSPVZ is modulatory in nature^28^.

Consistent with previous reports demonstrating that melatonin modulates light-induced autonomic responses^71^, inhibits single-unit SCN neuronal firing in vitro, and antagonizes light-induced SCN field potentials (our study) and 2-deoxy-[1-14C] glucose uptake in the SCN^72–74^, we show that exogenous melatonin inhibits light-induced cFos expression in the SCN. According to the SCN-vSPVZ communication concerning sleep^75^, melatonin is expected to potentiate light-mediated activation of the vSPVZ by inhibiting SCN neuronal firing, which our data supports. We therefore propose that melatonin facilitates sleep induction by regulating SCN activity, hence modulating the circadian wake-promoting signal. Therefore, the effect of endogenous melatonin on sleep timing is conserved across diurnal and nocturnal species. Moreover, the nighttime neurohormone melatonin has sleep-promoting properties in night-active rodents.

The tetrapod ancestor that gave rise to today’s diurnal mammals was probably a nocturnal creature^76^. It is unknown whether this ancestor was melatonin-proficient, but it is highly likely since melatonin is present in almost all animal species. Its function as a cellular protectant against free radicals and oxidation goes back around 3.6 billion years. The various functions of melatonin reported within a single mammalian species are most likely inherited from their ancestors during evolution. One theory proposes that the diversification of melatonin function occurred more rapidly than the evolution of new molecules. Melatonin’s most recently evolved function is sleep promotion by inhibiting alertness. However, the role of melatonin in sleep has primarily been studied in diurnal species, likely due to the anti-phasic relationship between the melatonin rhythm and sleep in nocturnal species. Here, we show that the function of melatonin in facilitating sleep, likely by inhibiting the signal for wake, is evolutionarily conserved. Testing our findings on the proposed model by Diessler et al.^28^, explaining the evolutionary switch that creates the anti-phasic relationship between diurnal and nocturnal mammals, we found that the addition of melatonin does not change the model’s outcome in sleep regulation (Fig. 7).

Lastly, the structure of the sleep-wake cycle, defined by the vigilance states of sleep and wake, shapes daily rhythms in physiology, including day-night differences in local hippocampal signaling, as shown here. Such considerations are often overlooked in basic preclinical and clinical study designs. It is thus advisable for comparison studies to adjust the timing of sampling or behavioral assessments based on differences in the sleep-wake rhythm.

## Methods

### Animals

In this study we used adult male (6–7 months old) mice of the C3H/HeN strain that are pineal melatonin proficient and express melatonin receptors 1 and 2 (MT_1/2_^+/+^) and age-matched C3H/HeN mice deficient for melatonin receptors (MT_1/2_^−/−^). Additionally, we used C57BL/6 mice that lack an appreciable endogenous melatonin rhythm^21,23,77^ but have functional MT receptors^78–80^. Thus, C57BL/6 mice endow a retained capacity to interpret an exogenous melatonin signal as previously shown^81^ and were therefore used in experiments simulating the mid nighttime melatonin surge.

Mice, C3H/HeN-Pde6brd1 KO/mt1,2 (short: MT_1/2_^−/−^) were locally bred at The University of Queensland’s Biological Resources Facility. Mice deficient for both melatonin receptors (MT_1/2_^−/−^) were obtained by crossing MT_1_^−/−^^82^ and MT_2_^−/−^^83^ mice and breeding the MT_1/2_^−/−^ offspring for at least 10 generations^84^. The C3H/HeN mice used in this study were the non-transgenic littermate controls (short: MT_1/2_^+/+^). Mice were individually housed in circadian cabinets (Phenome Technologies Inc., IL, USA), with water and standard chow available *ad libitum*, in 12:12 h light:dark (LD) cycles (250 lux) unless stated otherwise. Information related to the light source in the circadian cabinets can be found here (https://actimetrics.com/products/circadian-cabinets/). The same light source was also used to light pulse the mice. Mice were allowed to acclimatize to the circadian cabinets for at least 1 week. All experimental procedures were approved by The University of Queensland’s Animal Ethics Committee and conducted in accordance with both the Australian code for the care and use of animals for scientific purposes, and the ARRIVE guidelines^85^. All experimental assessments and data analyses were conducted by investigators blinded to group allocation to prevent bias.

### Profiling nocturnal locomotor activity and metabolism

Adult male MT_1/2_^+/+^, MT_1/2_^−/−^, and C57BL/6 mice were used to assess the role of melatonin receptor signaling on the immobility/mobility-defined sleep/wake profile. Activity data (running-wheel usage or horizontal activity) were acquired and analyzed using ClockLab (Actimetrics, IL, USA) and rhythmic metabolic data (energy expenditure), including drinking behavior, were obtained using the Phenomaster (TSE Systems, Germany).

To assess the action of melatonin receptor signaling on the nocturnal activity profile, C57BL/6, and MT_1/2_^−/−^ mice were provided *ad libitum* access to melatonin in drinking water. Melatonin was dissolved in ethanol and diluted in drinking water to a final concentration of 0.02 mg/ml melatonin/0.1% ethanol as previously described^51,86,87^. Vehicle controls received ethanol in drinking water at a final concentration of 0.1% as previously described^51,86,87^.

### Photosomnolence (light-induced behavioral sleep)

Light induces behavioral quiescence at which time the mice assume a typical sleep posture. We confirmed using EEG (Fig. 1e-g) that wheel running and infrared beam-based activity suppression by light (LED; 400–700 nm wavelength) coincides with behavioral sleep, confirming previous reports^88,89^.

Mice, individually housed in RW cages and entrained to an 18:6 h LD cycle, were subjected to a cycling 3-day protocol^60^; a baseline day, a light-pulse day, and a recovery day. The duration of the applied LP was set to 3 h, and the illumination used was 1000, 500, 250, 125, 65, or 32 lux. The LP was applied 60 min after the light-to-dark transition. The sleep-inducing effect of light was assessed by comparing the total activity during the 3-h LP with the corresponding 3-h of baseline activity on the day prior (baseline day).

To test whether melatonin modulates photosomnolence, mice entrained to the 18:6 h LD cycle were habituated to the experimenter for 2 weeks. The treatment group was injected 30 min before the LP with melatonin (3 mg/kg, intraperitoneal—i.p.) dissolved in ethanol (0.05% ethanol in 0.9% saline) or a combination of melatonin and the competitive melatonin receptor antagonist luzindole (3 mg/kg, intraperitoneal); according to calculations and method of delivery^90,91^. Controls received vehicle only (0.05% ethanol in 0.9% saline). Intraperitoneal injections were used to ensure a rapid and acute drug responses. For high-throughput screening, immobility-defined sleep was measured using both video and infrared beam-break recordings.

### Sleep analysis using running-wheel recordings

A method to assess sleep is to monitor RW activity by registering wheel turns (Actimetrics, USA). The behavior was tracked continuously for the duration of the experiment and analyzed using ClockLab (Actimetrics, USA). Sleep was defined based on episodes of RW quiescence of 60 s or longer, as previously described^26^.

### Sleep analysis using infrared beam-break recordings

A method to assess sleep timing (onset, offset, and duration) is to monitor home cage activity using an infrared interruption counter system (TSE Systems, Germany). The behavior was tracked continuously for the duration of the experiment and analyzed using ClockLab (Actimetrics, USA) and the sleep analysis software (TSE Systems, Germany). SO was determined based on episodes of immobility of 60 s or longer, also known as a sleep bout^26^.

### Sleep analysis using video recording

To visually identify SO, offset, and sleep maintenance, mice were monitored using a day/night vision capable camera system (Rasperry Pi NoIR Camera Module v2, RS Components Pty Limited, NSW-Australia). Video data were acquired wirelessly at a resolution of 640 × 480 pixels at 30 frames per second. A frame-by-frame comparison every 200 ms was performed, with ≥1% change between frames registered as a movement. This sensitivity threshold (sampling rate of 5 Hz) allowed a reliable differentiation of true locomotor activity from changes in body posture and breathing during sleep. 60-s or greater of camera-defined immobility was identified as a sleep bout^26^.

### Sleep analysis using radiotelemetry

We used the HD-X02 radiotelemetry implants from Data Science International (DSI, USA) to measure EEG, electromyography (EMG), and activity rhythms (for details see ref. ^92^). Surgeries were performed under 2–3% isoflurane in 100% oxygen. Mice received subcutaneously 1 mg/ml meloxicam (TROY Laboratories P/L, NSW, Australia) post-operative for 3 days. The EEG electrodes were placed epidurally above the motor cortex (AP + 1, ML + 1 mm left from bregma) and the visual cortex (AP–3, ML + 3 mm right from bregma)^93^. EEG electrodes were fixed with glass ionomer cement (Kent Dental, Kent Express Ltd., UK). EMG electrodes were anchored in the trapezius muscle. The scalp was closed using nonabsorbable 6–0 suture material (Ethicon, USA). Male mice (*n* = 3/group, aged 6–7 months) were housed individually in 12:12 h LD conditions (250 lux) for 2 weeks to fully recover. EEG and EMG were recorded at a sampling frequency of 500 Hz and captured by the Ponemah 6.4 software (DSI, USA). EEG and EMG recordings were scored in 4-s epochs by the automated rodent sleep-scoring software, NeuroScore 3.0 (DSI, USA) as Wake, NREM, or REM. Sleep scoring was additionally supported through video surveillance and an expert assessor.

To validate immobility-defined sleep as a method to evaluate sleep parameters (duration and latency) as previously described^26,27^, we compared the three methods of immobility-defined sleep analysis including RW activity and horizontal activity with EEG-defined sleep data in statistical software R using the ggpubr package (Fig. S5).

### Intracerebral recordings to assess the effect of melatonin on light-induced neuronal activity

The procedure for deep intracerebral implantation in mice has been previously described^94^. Briefly, 12–16-week-old male mice weighing 25–35 g were deeply anesthetized with isoflurane (4% for induction and 1.5% for maintenance) and fixed in a stereotaxic frame. A midline incision was made on the head and down the neck to fully expose the parietal bones and the subcutaneous tissue. A tungsten microelectrode coated with parylene (14.5 length, 250 μm diameter, 50–100 kOhm impedance, epoxylite insulation microelectrodes, FHC Inc., USA) was inserted into the brain aimed at the SCN (coordinates: 0.4 mm posterior and 0.2 mm lateral to the bregma, 5.5 mm depth from the surface of the dura mater). An epidural reference electrode was placed on the dura mater above the cerebellum, and the inserted electrodes were fixed with glass ionomer cement (Kent Dental, Kent Express Ltd., UK). The intracerebral electrode to record the SCN neuronal activity was then connected to an HD-X02 radiotelemetry device implanted subcutaneously (DSI, USA) to transmit the neuronal activity in free-moving mice. The scalp was closed using nonabsorbable suture material (Ethicon, USA).

Animals were single-housed under a 12:12 h LD cycle (250 Lux) and allowed to recover from surgery for at least 2 weeks. Local field potential, physical activity and body temperature were acquired for 24-h. The data from the implanted electrode were collected at 500 Hz and captured by Ponemah 6.4 software (DSI, USA). A band-pass filter of 0.1–25 Hz was applied to the EEG signal using NeuroScore (DSI, USA), and the root mean square (RMS) per 10-s interval was calculated. Change in the power of the signal (%change) was determined by comparing the RMS of every 10-s bin of neuronal spikes during the LP with the average RMS of neuronal spikes for the 10 min prior to the time the light stimulus was introduced.

Electrode placement was verified postmortem. Briefly, mice were anaesthetized with isoflurane and the transmitter was disconnected from the implanted electrode. A small positive current was then passed through the recording electrode to mark the location within the brain. Brains were extracted and fixed in 4% paraformaldehyde. Serial coronal sections of brains were cryosectioned (20 μm thick) and electrode placement was histologically confirmed by staining with haematoxylin and eosin.

### Immunohistochemical analysis of light and melatonin on neuronal substrates of sleep

Mice housed in 18:6 h LD cycles were injected with either melatonin or vehicle 30 min prior to a 1-h light exposure (65 lux). Mice were transcardially perfused using 0.9% saline followed by 4% paraformaldehyde (PFA) in phosphate buffered saline (PBS). Control mice were perfused under dim red light. Extracted brains were post-fixed in 4% PFA overnight at 4 °C. Fixed brains were cryoprotected in sucrose solutions in PBS at 4 °C, then frozen and stored at −20 °C before cryosectioning into 16 μm coronal slices. For immunofluorescence, the sections were washed in PBS, subjected to antigen retrieval using citrate buffer (pH 6) and heating. The tissue was incubated in bovine serum albumin (BSA) in PBS and 0.3% Triton X-100 (PBST) (0.02 g BSA/mL PBST) for 1-h at room temperature, followed by an overnight incubation in primary antibodies. Markers to be stained included cFos, the immediate early proto-oncogene product (Santa Cruz Biotechnology, #sc-166940) as marker for neuronal activity^95^, and pCREB (Millipore, #06-519) as marker for memory processing in the hippocampus, diluted in PBST (1:1500) at 4 °C. The tissue was washed in PBST and incubated with anti-rabbit secondary antibody (1:1000, Invitrogen, #A-11078). Fluorescence mounting medium containing DAPI (Vectashield from Vector Laboratories, Australia) was used for sealing the tissue with glass coverslips and left to rest for 24-h at 4 °C before imaging.

Regions of interest (ROI) were imaged using a Leica DMi8 SP8 inverted confocal microscope and the Leica Application Suite X Software. 1024 × 1024-pixel images were captured using a 20x objective. An average of 15 images per field-of-view were acquired as z-stacks (step size of 1.75 µm), and the five center-most images were used for analysis. Laser exposure, detector gain, pinhole and offset were kept constant across all samples.

### Quantification and statistical analysis

The Imaris 8.4.1 software (Bitplane) was used to process the images. cFos-positive nuclei were identified by co-localization of cFos and DAPI staining exceeding Imaris-defined automatic-threshold background subtraction. The total number of cFos positive nuclei was normalized to the volume of the ROI. Statistical analyses were performed using GraphPad Prism (La Jolla, CA, USA). Data are presented as means ± standard error of the mean (SEM) as indicated in figure legends. Student’s *t*-Test was used to determine statistical significance between treatments. One-way ANOVA with Tukey’s post-hoc analysis or one-way ANOVA followed by pairwise *t*-tests were used for time series. Multivariate experiments were analyzed using a two-way ANOVA with Tukey’s post-hoc analysis. CircaCompare was used to estimate and statistically support differences in MESOR, amplitude, and phase, between diurnal sleep/wake rhythms^96^ *p* values are indicated in figure legends. The sample sizes were determined using a power analysis in GraphPad Prism, designed to detect a minimum of 2-fold difference in the measured parameters with 80–85% power, based on an alpha level of 0.05. Unless stated otherwise, no criteria were applied for excluding data points. All experimental groups were randomly assigned to ensure unbiased allocation of subjects. This randomization process was applied to all aspects of the study, including treatment administration and data collection, to minimize potential confounding variables and ensure the validity of the results.

## Supplementary information

Supplementary Materials

## Data Availability

The datasets used and/or analyzed during the current study available from the corresponding author on reasonable request.
