# Supplementary material for: Melatonin’s role in the timing of sleep onset is conserved in nocturnal mice
Source: NPJ Biol Timing Sleep. 2024 Nov 1;1:13. doi: 10.1038/s44323-024-00013-1 (PMC11530376; doi:10.1038/s44323-024-00013-1)
Supplement: Supplementary file 1 — Supplementary Materials [file 44323_2024_13_MOESM1_ESM.docx]

**Supplementary Materials for**

***Melatonin’s role in the timing of sleep onset is conserved in nocturnal mice.***

**Authors:** Pureum Kim^1^, Nicholas Garner^1^, Annaleis Tatkovic^1^, Rex Parsons^1#^, Prasad Chunduri^1^, Jana Vukovic^1,2^, Michael Piper^1,2^, Martina Pfeffer^3^ Marco Weiergräber^4^, Henrik Oster^5^ and Oliver Rawashdeh^1*^

**Affiliations:** *1. School of Biomedical Sciences, Faculty of Medicine, University of Queensland, Brisbane, Australia.* *2. Queensland Brain Institute, University of Queensland, Brisbane, Australia. 3.Center for Anatomy and Brain Research, Institute for Anatomy 2, Düsseldorf, Germany. 4. Experimental Neuropsychopharmacology, Federal Institute for Drugs and Medical Devices, Bonn, Germany. 5. Institute of Neurobiology, Center of Brain, Behavior & Metabolism, University of Lübeck, Lübeck, Germany*

* Correspondence to Oliver Rawashdeh ([o.rawashdeh@uq.edu.au)](mailto:o.rawashdeh@uq.edu.au))

**
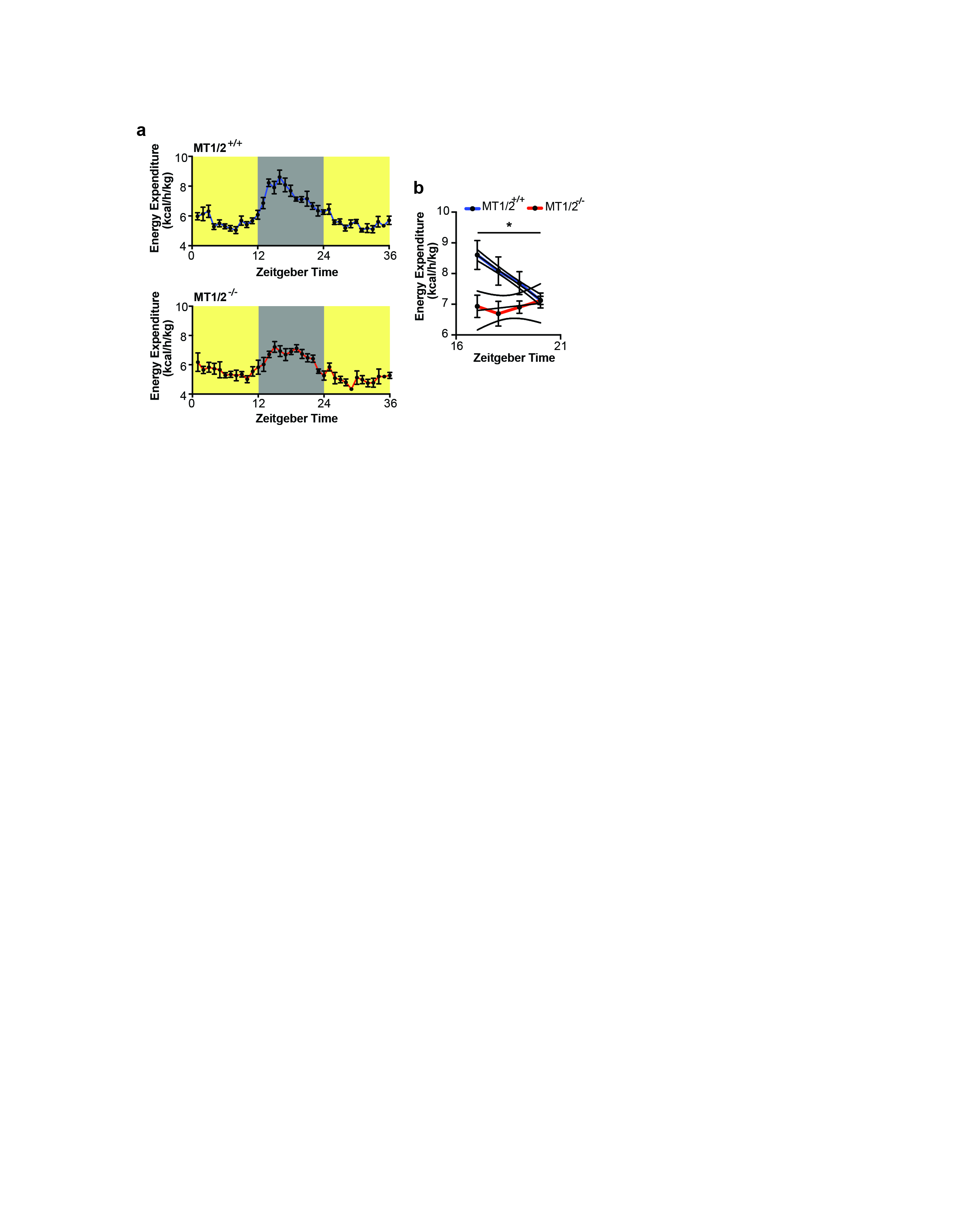
**

**Fig. S1:** (**a**) Diurnal profiles of energy expenditure in MT_1/2_^+/+^ (*n*=5) and MT_1/2_^-/-^ (*n*=4) mice. (**b**) Hourly averages in energy expenditure during the 2^nd^ half of the dark phase (ZT17-20) in MT_1/2_^+/+^ (blue line) and MT_1/2_^-/-^ (red line) mice. Statistical significance within genotype was determined using one-way ANOVA (p<0.05). * Indicates p <0.05. The data are presented as means ± S.E.M.

**
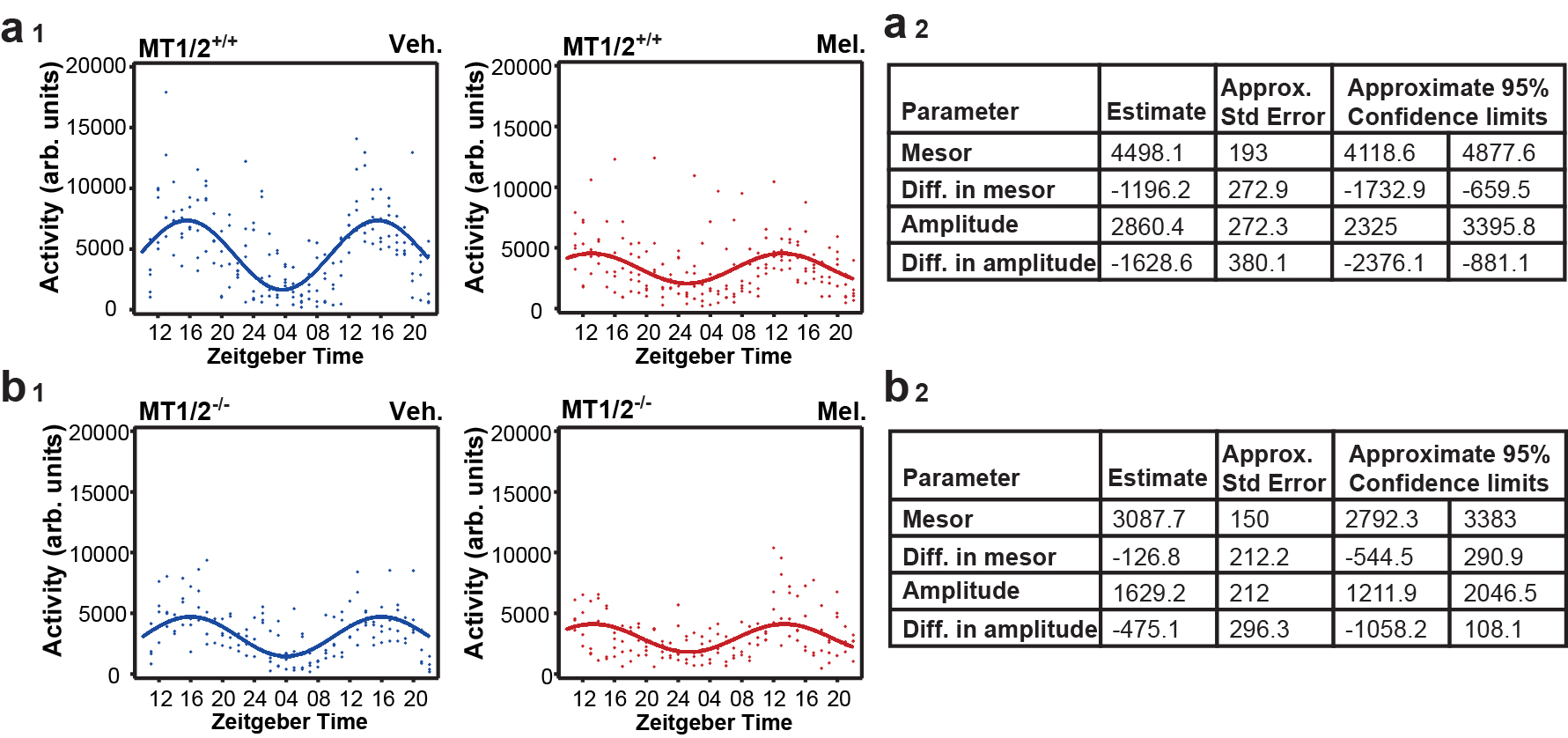
**

**Fig. S2:** (**a_1_**, **b_1_**) Diurnal activity profiles for MT_1/2_^+/+^ (*n*=5) and MT_1/2_^-/-^ (*n*=4) mice treated with either vehicle (veh.) or melatonin (mel.) in drinking water. (**a_2_**, **b_2_**) Tabulated results from statistically comparing the mesor and amplitude for the diurnal activity rhythms of veh. and mel. groups using CircaCompare.

**
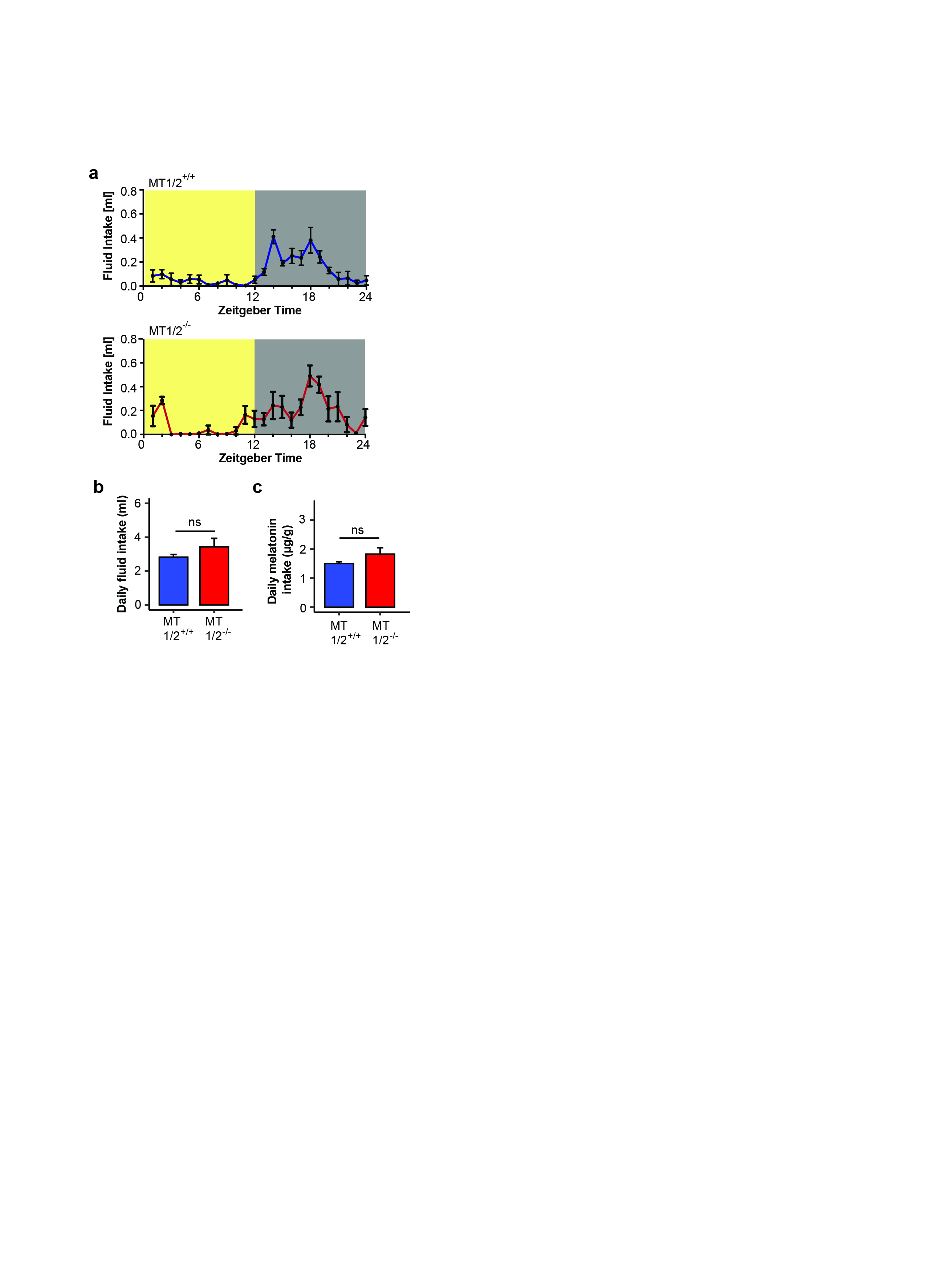
**

**Fig. S3:** (**a**) 24 hour diurnal drinking profiles for MT_1/2_^+/+^ (*n*=5) and MT_1/2_^-/-^ (*n*=4) mice, acquired using metabolic cages. The drinking water was supplemented with melatonin. (**b**) Comparison of the total amount of daily fluid intake by drinking and (**c**) daily melatonin intake normalized to their body weight between MT_1/2_^+/+^ and MT_1/2_^-/-^ mice. (Student’s *t*-Test, *p*<0.05). ns indicates *p*>0.05. The data are presented as means ± S.E.M.

**
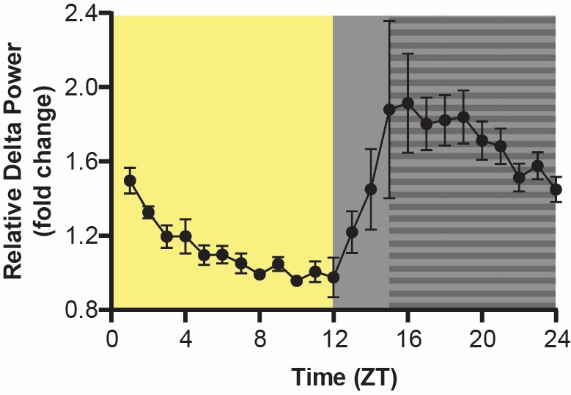
**

**Fig S4:** 24 hour profile of the normalized delta power during NREM sleep for MT_1/2_^+/+^ (*n*=6) mice. The delta power in the frequency range of 0.5-4.0 Hz restricted to NREM sleep for each hour was normalized to the average delta power between ZT8 and ZT12. The shaded area refers to nocturnal rises in the plasma level of melatonin in mice from previous literatures^1,2^. The data are presented as means ± S.E.M.

**
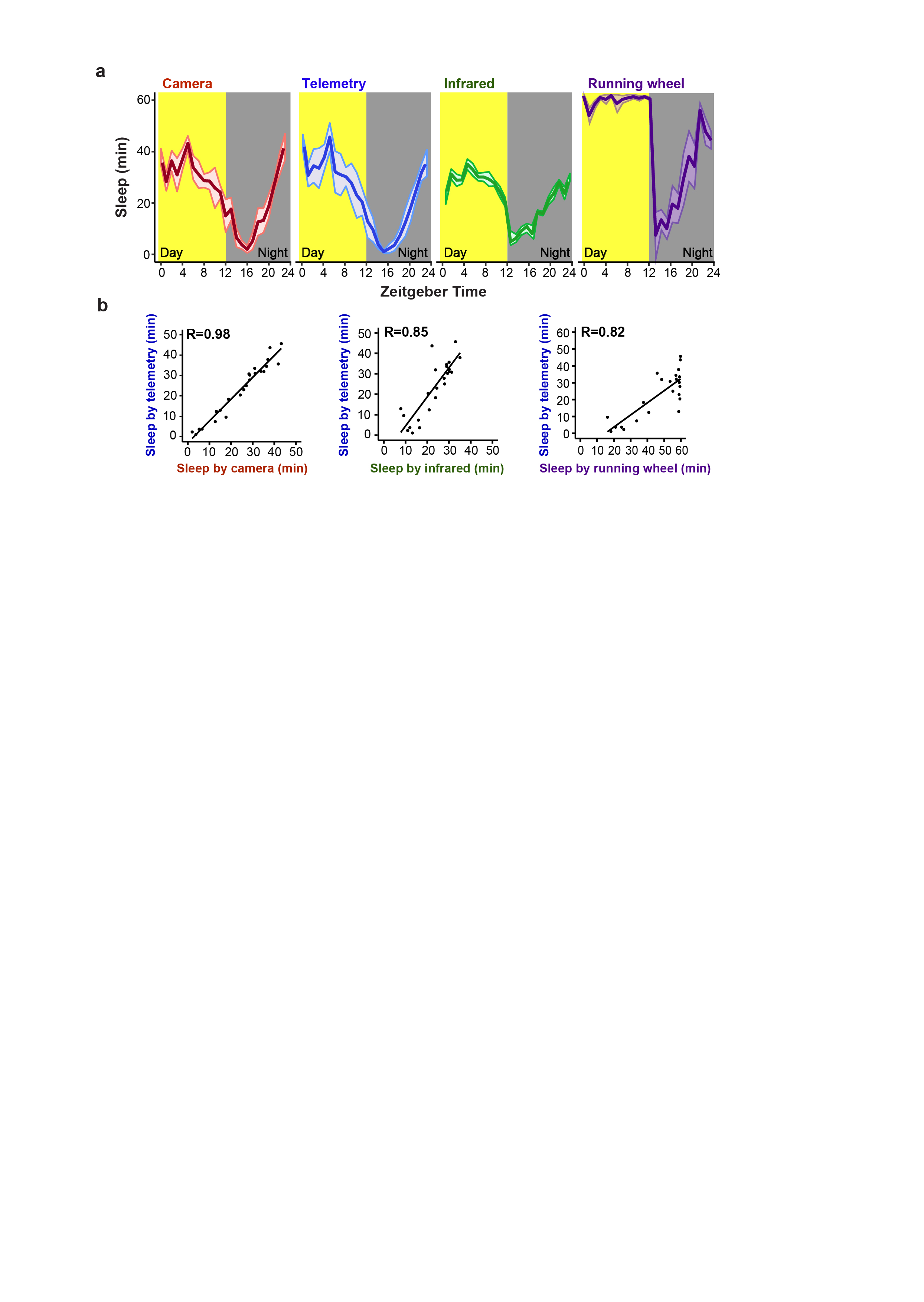
**

**Fig. S5:** (**a**) Illustrated are the day-night distribution and the amount of sleep determined by different activity-dependent data acquisition methods (camera-based, red, infrared-based; green running wheel; purple) and telemetry (electroencephalography; blue). (**b**) Shows the correlations between the different high-throughput data acquisition methods (camera, infrared and running wheel) and the gold standard electroencephalography measurement of sleep amount.

**References**

1. Kasahara T, Abe K, Mekada K, Yoshiki A, Kato T. Genetic variation of melatonin productivity in laboratory mice under domestication. *Proc Natl Acad Sci U S A*. Apr 6 2010;107(14):6412-7. doi:10.1073/pnas.0914399107

2. Christ E, Pfeffer M, Korf HW, von Gall C. Pineal melatonin synthesis is altered in Period1 deficient mice. *Neuroscience*. Dec 1 2010;171(2):398-406. doi:10.1016/j.neuroscience.2010.09.009
